# Determinants of stakeholders’ attitudes towards biodiesel

**DOI:** 10.1186/s13068-017-0908-8

**Published:** 2017-09-15

**Authors:** Latifah Amin, Hasrizul Hashim, Zurina Mahadi, Maznah Ibrahim, Khaidzir Ismail

**Affiliations:** 1 Pusat Citra Universiti, Universiti Kebangsaan Malaysia, 43600 UKM Bangi, Selangor Darul Ehsan Malaysia; 2 Faculty of Social Sciences and Humanities, Universiti Kebangsaan Malaysia, 43600 UKM Bangi, Selangor Malaysia

**Keywords:** Attitude, Biodiesel, Determinants, Structural equation modelling, Malaysia

## Abstract

**Background:**

Concern about the inevitable depletion of global energy resources is rising and many countries are shifting their focus to renewable energy. Biodiesel is one promising energy source that has garnered much public attention in recent years. Many believe that this alternative source of energy will be able to sustain the need for increased energy security while at the same time being friendly to the environment. Public opinion, as well as proactive measures by key players in industry, may play a decisive role in steering the direction of biodiesel development throughout the world. Past studies have suggested that public acceptance of biofuels could be shaped by critical consideration of the risk–benefit perceptions of the product, in addition to the impact on the economy and environment.

**Results:**

The purpose of this study was to identify the relevant factors influencing stakeholders’ attitudes towards biodiesel derived from crops such as palm oil for vehicle use, as well as to analyse the interrelationships of these factors in an attitude model. A survey of 509 respondents, consisting of various stakeholder groups in the Klang Valley region of Malaysia, was undertaken. The results of the study have substantiated the premise that the most important direct predictor of attitude to biodiesel is the perceived benefits (*β* = 0.80, *p* < 0.001). Attitude towards biodiesel also involves the interplay between other factors, such as engagement to biotechnology, trust of key players, attitude to technology, and perceived risk.

**Conclusion:**

Although perceived benefit has emerged as the main predictor of public support of biodiesel, the existence of other significant interactions among variables leads to the conclusion that public attitude towards biodiesel should be seen as a multi-faceted process and should be strongly considered prior to its commercialisation.

## Background

Biofuels or bioorganic fuels can be classified into first, second, third, and fourth generation. First-generation biofuels are mainly derived from edible sources such as sugar cane, maize, wheat, and palm oil, second-generation biofuels are derived from non-food crops such as *Jatropha miscanthus* and lignocellulosic feedstocks such as energy crops, cereal straw, and forest residues, third-generation biofuels are made from algae, whereas fourth generation include photobiological solar fuels and electrofuels [1]. As the first-generation biofuels are derived from food crops, one immediate advantage is that the infrastructure for planting, harvesting and processing is already in place [2]. However, the main concern is food security. The second-generation biofuel, which is also known as advanced biofuel, is better in terms of return values, but requires greater production processes than the first-generation biofuel, especially in respect of lignocellulosic feedstock [2]. The third-generation biofuel, which is derived from algae, is currently the most productive feedstock, as algae produces outstanding yields and is easier to process compared to the first-generation and second-generation biofuels [2]. However, the cost of algae biofuel is much higher due to its need for water and fertiliser. In addition to oil-producing algae, several species of yeast and filamentous fungi have the capability to synthesise lipids in their cells which provide the raw materials for biodiesel production [3]. The fourth-generation biofuels such as photobiological solar fuels and electrofuels are expected to bring fundamental breakthroughs in the field of biofuels [4]. The technology for the production of such solar biofuels is based on direct conversion of solar energy into fuel using raw materials that are inexhaustible, cheap, and widely available.

The differentiation between the biofuels is critical for the stakeholders, due to the possible economic, social, and environmental impacts. Each generation of biofuel has its own economic implications as the source, production process, distribution, and markets are different. The first-generation biofuels may affect the social aspect, especially food security, as they are derived from food crops. The second-generation biofuels, which are derived from plant dry matter or lignocellulose, may not impact food security directly, but seed crops may compete for fertile land with food crops. There are also concerns on land use changes that might lead to ecological damage for the first-generation and second-generation feedstocks. The third-generation biofuels, which are derived from algae, may not pose any social and economic risks, unlike the first and second-generation biofuels, but pose a risk to the environment. The major drawback of algae production is that it requires a large amount of fertiliser. This produces greater greenhouse gas emissions in the production, which surpasses the amount saved by using algae-based biofuel [2]. In addition, fertilisers may pollute the water and soil significantly [5]. The technology related to the fourth generation of biofuel seems the most promising however, is still at the level of basic research. These tangible and intangible effects of different generations of biofuels should be taken into account by stakeholders before they venture into the industry.

Biodiesel is an alternative renewable liquid fuel that can be used in any diesel engine without prior modification and has become more attractive recently because of its environmental benefits [6]. It reduces exhaust emissions of unburned hydrocarbons (HC), carbon monoxide (CO), sulphates, polycyclic aromatic hydrocarbons, nitrated polycyclic aromatic hydrocarbons, and particulate matter (PM) compared to conventional petroleum diesel engines [6]. Biodiesel is produced from raw vegetable oil or animal fat and methanol. Most of the biodiesel developed nowadays originate from soybean oil, methanol, and an alkaline catalyst [6]. It is chemically distinct from petroleum diesel and bears quality risks depending on feedstock, physical properties, and the production process. In terms of sulphur content, aromatic content, and biodegradability, biodiesel is a better alternative compared to conventional diesel fuel [7]. In Malaysia, initiatives to develop ethanol and other biofuels from non-food agricultural crop sources such as *Jatropha curcas* and palm oil biomass are still progressing [8]. Commercial production of biodiesel using palm oil feedstock produced 833 million litres of biodiesel in 2016 [9]. The biofuel-fossil fuel substitution measure is very timely for Malaysia, as industries, especially manufacturing activities, have dominated economic activities in recent years [10]. The energy consumption for petroleum products in this country has shown a dramatic increase from 5550 ktoe in 1980 to 9825 ktoe in 1990 and to a further increase of 19,581 ktoe in the year 2000, but gradually became more consistent from 2000 to 2010 (24,403 ktoe) and onwards. The average annual growth rate of fossil energy consumption is forecast at 0.8% until 2025. Among the available petroleum products, motor petrol and diesel have the greatest consumption [11].

Malaysia’s oil reserves have been maintained between 3 and 4 billion barrels since 1985 [12], which is estimated to be sufficient for domestic consumption until 2027, even without new discoveries and imports indicating that there is no immediate energy security issue in Malaysia. Malaysia does not consume oil entirely from its production but exports high-grade oil and imports lower grade oil [12]. The volume of exported petroleum has consistently exceeded the volume of imported petroleum since the year 2000, and as in 2014, the volume of exported and imported petroleum is 16,009 and 10,399 kilotons, respectively [11]. Although the oil reserves of this country are projected to be sufficient for domestic use until 2027, a new source of renewable energy should be empowered immediately for future energy security, to reduce dependency on imported oil and to reduce dependency on fossil oil. The National Biofuel Policy, enacted in March 2006, envisions the use of sustainable and viable sources of energy to reduce the dependency on fossil fuels [13, 14]. Furthermore, the Malaysian Biofuel Industry Act 2006 contains the mandatory provision that diesel fuel production in Malaysia must be mixed with 5% biodiesel to make use of the abundant supply of palm oil in the country. In practice, the national blend rate in 2016 was 7.0% and is forecast to increase to 10% in 2017 [9].

Malaysia has been recognised as a highly developing country with great potential to become a developed country by 2010, as outlined in the Vision 2020 [15]. Although the Malaysian economy is growing steadily with its income highly dependent on energy resources such as crude oil and natural gas, there is a need to diversify its economic structures and increase productivity to be able to achieve the developed country status. Biodiesel production has the potential to contribute to further development of Malaysia from a highly developing country to a developed country. In 2016, Malaysia registered a commendable growth of 4.2% with a GDP value of Malaysian ringgit (MYR) 108.2 billion, powered by the three sectors of services, manufacturing, and construction. The GDP from agriculture decreased to MYR 21,787 million in the first quarter of 2017 from MYR 23,095 million in the fourth quarter of 2016. Despite the reduction, this sector has overtaken the construction sector in Malaysia’s economy due to strong growth in palm oil and rubber. Palm oil has become the major contributor in the agricultural sector to Malaysia’s GDP from 2011 to 2015 [10], and is expected to dominate the agriculture share of GDP in the future. The volume of people in employment in Malaysia from 2015 to 2017 is approximately 45% of the total population, with 12.5% working in the agricultural sector. Since palm oil is the principal agricultural crop in Malaysia, it is expected that the majority of those people work in this sector. The GDP per capita in Malaysia has steadily risen from 2010 to 2016. At US$11,028.20, the GDP per capita in 2016 is equal to 87% of the world average [16]. The relationship between GDP per capita and the employment rate has always been positive [17]. The expansion of biodiesel production in Malaysia could spur socioeconomic growth and development through the provision of more job opportunities and eventually higher GDP per capita, social infrastructural development, and better living standards for Malaysians [18]. To this end, the Malaysian government has established modalities to ensure that biodiesel becomes a major supplier of energy fuels for the transport and industrial sectors of the economy. To reiterate its commitment, the Malaysian government has earmarked over MYR 300 million in grants to the biodiesel industry, primarily to fund the infrastructural development of bio-refining facilities for blending and upgrading biodiesel at 36 existing petroleum diesel depots around the country [18]. This will support investor confidence in the biodiesel industry by aiding the development of more biodiesel plants, creating more jobs, and improving the country’s infrastructure.

Biodiesel does have some disadvantages. It is poor in quality and efficiency in comparison to regular diesel or gasoline-based fuel [19]. Its lower energy output requires greater quantities for performance. This has resulted in the reduction of the cost effectiveness of biodiesel in comparison to fossil-based diesel [20]. Furthermore, the limited amount of biodiesel produced, due to the small numbers of producers, has reduced market competition and increased prices. At present, there is an inconsistent supply of feedstock and high processing costs, which have also contributed to the higher price of biodiesels in comparison to fossil-based diesel [2]. Additionally, the saturated fatty acids in the biofuel gel in cold weather and form crystals that plug filters. Furthermore, long-term storage of biodiesel is prone to degrade gaskets and seals [21]. Biodiesel also has a higher affinity toward moisture compared to petroleum diesel, and the water-retaining capacity of biodiesel is higher than diesel which can cause problems such as water accumulation and microbial growth in fuel tanks and transportation equipment [22]. The acidity of biofuel feedstock especially vegetable oil could result in incomplete transformation of oil to biofuel which eventually would affect the engine performance [23]. In terms of capability, car engines manufactured before the year 1994 have to be modified to be compatible with biofuel due to their rubber pipe components [24]. Most of the car engines and fuel system produced afterwards are compatible with biodiesel mixed with fossil fuel up to 20% blend but not the pure biodiesel. Any remaining problems from the use of biodiesel will be expected to be resolved in stages through research.

Apart from the performance aspect, interest in the use of vegetable oils for biodiesel production is also a disadvantage, as it may lead to the potential exhaustion of food stock [25], due to the fact that global biodiesel production is mainly derived from rapeseed oil, soybean oil, palm oil, sunflower, and other feedstocks [26]. In certain circumstances, biodiesel seems to be environmentally friendly; however, it might also be socio-economically hazardous [27]. It is believed that a rapid growth in biodiesel production may take up precious agriculture land, affecting food security and subsequently causing food prices to increase [28–32]. There has been a debate about biofuels as a possible major source of food prices rises which lead to food crises [33]. In his analysis, Rathman [34] noticed that the dynamics of land use worldwide have changed, with the large-scale production of agro-energy, which has caused a shift of areas traditionally allocated to food production to biofuels. This scenario might have led to an increase in food prices in the short term, but this phenomenon is not yet significant. On the other hand, others argued that the land dedicated to biofuels was marginal and has been compensated by the increase in agricultural productivity [35]. There are also suggestions to use feedstocks from non-crop sources, such as waste or the by-products of existing processes, in order to break the relationship between food supplies and biofuel [36].

In terms of environmental impact, economic model estimates for land use change (LUC) associated with food-based biofuels showed that the biofuels contribute to greater greenhouse gas emissions [37]. LUC can be a factor in the atmospheric concentration of CO_2_ (carbon dioxide), which in turn results in climate change [38]. In fact, the total LUC carbon intensity of bioethanol was found to be from −29 to 384% of gasoline, suggesting that LUC could potentially alter the greenhouse gas emissions’ benefits of biofuels [39]. Furthermore, due to the expansion of agricultural land for biodiesel production, it is estimated that biodiversity has been reduced by 60% in US corn and soybean fields and by 85% in Southeast Asian palm oil plantations compared to unconverted habitat [37]. Biodiesel production may also face the vulnerabilities situation including equity issues or loss of family farms [40].

The total land area in Malaysia is 32.98 million hectares of which 31% is arable [41]. Agriculture has been identified as one of the drivers of economic activity in the 10th Malaysian Plan to steer Malaysia to become a high income nation by 2010. Additionally, the palm oil industry has been recognised as the fourth largest contributor to the national economy [42]. Table 1 demonstrates the areas of cultivation of the main agricultural crops in Malaysia. There appears to have been a significant increase in the palm oil area from 1987 to 2015, the paddy area is stable, but there has been a steady decline in hectares used for rubber, coconut, and cocoa. In 2015, the production of paddy has increased 16.6% from the previous year, which covered 730,000 ha in area. The planted area of palm oil also increased 4.6% (5,642,900 ha) while the rubber planted area has reduced from 0.4% to 824,000 ha [10]. The wetland paddy is cultivated in areas where palm oil cannot be grown, so there is no issue of land substitution between paddy and palm oil. The cultivation of dry paddy in the highlands only represents 10% of paddy plantation [43]. The increase in the palm oil cultivation area was more likely to have been at the expense of coconut, cocoa, and rubber. The replacement of these crops by palm oil was due to the drop in price of these commodities, especially rubber, which has forced the farmers to move to palm oil. It should be highlighted that paddy is a staple food in Malaysia, but coconut and cocoa is not, so the replacement might be more acceptable in terms of food security. Othman and Jafari [43] also reported an increase of land use for the cultivation of other agricultural crops including major vegetables, fruits, and pepper although there was a slight decline in the hectarage of sugarcane, coffee, and tea. Malaysia is considered to have low vulnerability with regard to food security, as the local production of basic food items, such as rice, fruit, vegetables, fish, and poultry, is adequate, while food supply and distribution is well organised [44]. Palm oil production in Malaysia in the year 2011/12 was 18.20 million tons which exceeded domestic consumption of only 3.42 million tons. Consequently, there was an excess of palm oil for local consumption [45]. The majority of the palm oil grown in Malaysia (16.6 million tonnes) is exported to other countries. In this case, the use of palm oil as a biofuel can actually provide social and economic advantages in developing countries by generating income for the needy to buy food. This has been well studied in the case of *Jatropha* as a biofuel source in Namibia [5].

**Table 1:** Land use pattern of main agricultural crops in Malaysia (1000 ha). Source: Department of Statistics, Malaysia 1987–2015

Crops	1987		2016	
	Planted area	Share with respect to total agriculture land area (%)	Planted area	Share with respect to total agriculture land area (%)
Rubber	1881.3	29.4	824.4	10.9
Palm oil	1640.2	25.7	5642.9	74.8
Coconut	320.6	5	82	1.1
Cocoa	370	5.8	21.7	0.3
Paddy	644.8	10.1	730	9.7

Globally, with the advent of various current technologies and the opening of new lands, the world can produce more food than is needed [41]. Various efforts have been made to develop crop species that can grow in harsh areas [46], as well as to develop pest and herbicide-resistant varieties [47], high-yielding crops and automated agricultural practices that could increase food production [41]. The land and technology are not the bottlenecks of volume. It should also be noted that food security is a multi-faceted issue that is not solely due to agro-land use. In reality, economics regulates the food market. Food–biofuel competition on a global level is an oversimplified approach. In regions where the amount of food produced exceeds the amount required, biofuel provides social and economic advantages rather than disadvantages. By channelling the excess foods to biofuel, food will not be wasted and income will be created for the people in need, who were originally unable to pay for food. From the economic perspective, the unlinked or linked market between food and biofuel has an opposite effect on food prices [48]. In the unlinked market, where the competition between food and biofuel is absent, any changes regarding food production including land use changes, will not affect the price of food. On the other hand, in the linked market, where the competition between food and biofuel is present, the food price will increase. The price increase has a positive social impact as it will benefit the farmers in terms of income. Starvation and wastage of food, overproduction and lack of food, are present at the same time, globally, sometimes even in the same region. It has been reported that almost one-third of total food production is discarded as food waste along the chain from farms to processing plants, retailers, food-service operations, marketplaces, and people’s kitchens [49, 50]. This wasted food is not delivered to people who need it but cannot pay. In this scenario, using food for biofuel is seen to be among one of the best solutions to resolve the issue of starvation and food wastage simultaneously. Ultimately, nobody cultivates land and spends money without the potential to make a profit.

Public perceptions about the opportunities and risks from the introduction of any new product into the marketplace are considered to be a key factor in avoiding market failures [51]. Thus, it is essential, when measuring public perceptions, to take into account the knowledge, opinion, and attitude of each of the dynamic stakeholder groups, whether directly or indirectly impacted by the industry [51]. This is due to the fact that public attitudes towards any technology reflect an individual’s belief in the ability of technological progress to solve the world’s problems in the future [52]. Consequently, it is important to understand an individual’s attitude towards any science and technology that has been stigmatised as inherently dangerous [53]. The concept of risk perception appeared on the stage of policy and has become a very important topic. Risk perception is implicated as the main determinant of public opposition to technology. Generally, the general public are relatively unfamiliar with biomass energy, which explains their lukewarm support of bioenergy. Their support or opposition to bioenergy depends on many factors, such as their knowledge and opinion of various attributes, demographics, their experience with renewable energy in the past, and their exposure to the mass media [51]. Studies of public perceptions of biodiesel have been somewhat limited, but nonetheless, to a certain degree, public beliefs about biofuels’ economic and environmental impacts were most important in shaping these opinions, rather than concerns about energy independence [54]. Clearly, the public are the major users of fossil fuels in the transportation sector and their willingness to switch to biodiesel is important to ensure the success of biodiesel production. Many of the citizens of developing countries have low environmental awareness and are not familiar with the operation of biofuels [55]. Some studies have revealed that the public have pledged their support for biodiesel [56]. But, nevertheless, many consumer stakeholders were sceptical that the bioenergy industry can create any significant economic impact on development in rural areas [51]. The majority of US farmers would use biodiesel if the prices were comparable to conventional diesel [57], whereas the American public agree that using biofuels is a good idea [58]. According to Eurobarometer, in 2010 more that 70% of Europeans agreed that biodiesel should at least be encouraged, adding that they are even more optimistic about more sustainable biofuels [28]. In 2011, Amigun et al. reported that biodiesel production and its supply chain may be affected by personal, social, and institutional factors and beliefs within different communities in South Africa [59].

In order to determine the optimal provision of biodiesels from a social point of view, it is necessary to combine both approaches, focusing on the advantages and shortcomings of biodiesel production and its relation to agricultural feedstock and food prices, as well as focusing on the social attitudes and opinions towards biodiesel and public opinion about its relationship to the increase in food prices [60]. This shows the importance of a study regarding public attitude towards biodiesel, as well as investigating the predictors which can influence and trigger specific attitudes towards biodiesel. Although there have been a number of studies examining public attitude towards biofuels throughout the world [54, 56, 59, 61], there has been limited research focused on the factors influencing public attitude towards biodiesel. Therefore, the present study has been undertaken to assess the Malaysian attitude towards biodiesel (primary biofuel) derived from crops such as palm oil to be used as biofuel for vehicles and to identify the factors that influence their acceptance of biodiesel using the structural equation model.

## Theoretical framework and hypotheses development

**Fig. 1:**
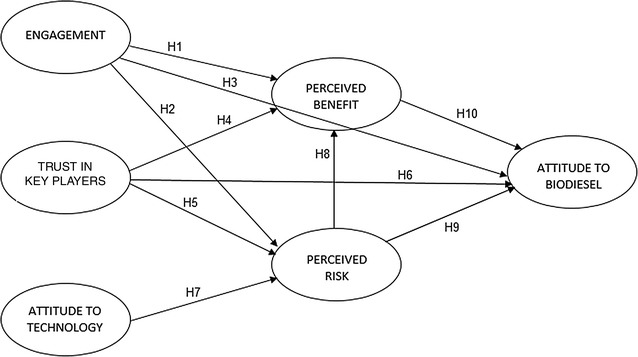
Research framework of public attitude towards biodiesel

Figure 1 represents the conceptual research framework of public attitude towards biodiesel labelled with the corresponding research hypotheses. The construction of this theoretical framework was designed based on previous attitude models and the inclusion of important factors that significantly influence attitude towards science and technology, as well as biotechnology-related applications from our earlier studies [62–67]. The findings from many established studies reviewed by Master and Resnik [67] concluded that public acceptance of science and technology is not fully dependent on science literacy or knowledge, but is influenced by people’s worldviews, concepts of risks and benefits, and trust in various social institutions including scientists, industry, and government. The variables selected and the proposed items were sent to five experts from various fields including attitude and perception studies, biotechnology and policy, environmental management, and measurement for validation. The model proposed in this study is fundamentally rooted in the original concept of Fishbein’s Multi-attribute Attitude Model [65]. The model begins with potential causes that are known to affect attitudes and the variables are arranged according to their assumed influence on the subsequent variables. Using the data from a pilot study, correlation analyses were carried out at a bivariate level to determine the relationships between the factors which were later used to develop the hypotheses (Table 2) [68]. The magnitude of the association between two variables was later determined by the regression weights from structural equation modelling (SEM) analysis. In general, the attitude towards biodiesel is determined by the specific perceptions of risks and benefits [69, 70], including issues related to food security [28]. General attitudinal factors such as engagement, trust of the key players, and attitude to technology are also included since past research has shown significant causal interpretations of risk and benefit perceptions resulting from these factors [66, 69, 71–73].

**Table 2:** The correlation matrix among factors in the research model attitude toward biodiesel

	Engagement	Trust of key players	Attitude to technology	Perceived benefit	Perceived risk	Attitude to biodiesel
Engagement	1					
Trust in key players	0.222**	1				
Attitude to technology	−0.158**	0.176**	1			
Perceived benefit	0.211**	0.316**	0.028	1		
Perceived risk	−0.157**	0.112*	0.377**	−0.160**	1	
Attitude to biodiesel	0.246**	0.338**	0.038	0.584**	−0.113*	1

* *p* < 0.05; ** *p* < 0.01

### Engagement

The variable engagement in this study consists of three sub-factors which are past and intended behaviour, awareness, and knowledge. The engagement concept was introduced by Gaskell et al. [72] based on a public issue taken from political science. Amin et al. also included past and intended behaviour, awareness, and knowledge as part of the focus in their study—these three components have been combined to form a variable called “engagement”, referring to the informed public [66]. Previous studies show that engagement in biotechnology was positively associated with encouragement of biotechnology applications and other positive predictors such as perceived benefits [63, 66, 72, 74]. Focus group research conducted in central Indiana found that despite being fairly knowledgeable about biofuel technology, as well as uninformed about biofuels policies, a narrow majority was supportive of biofuels in general, but expressed greater enthusiasm about “second generation” of biofuels [54]. On the other hand, Cacciatore et al. found that more knowledgeable respondents see fewer benefits of biofuels relative to the risks [56]. Frewer et al. also argued that acceptance of modern biotechnology by the public may not be related to awareness [75, 76].

Due to the importance of this factor in the public perception study, the following hypotheses were proposed for the association between engagement and other factors:

H1: When the stakeholders have higher engagement in biotechnology, then they will perceive higher benefits associated with biodiesel.
H2: When the stakeholders have higher engagement in biotechnology, then they will perceive lower risks associated with biodiesel.
H3: When the stakeholders have higher engagement in biotechnology, then they will have a more positive attitude towards biodiesel.

### Trust in the key players

The public cannot directly assess the benefits and risks of products and they have to rely on information provided by industry experts. Therefore, consumers have to depend on experts and institutions in the management of risks associated with technologies [77]. Gaskell et al. found a matrix of variables, including interest in aspects of the public domain, such as confidence in industry, regulation, and other civil society groups [72]. Amin et al. also stated that confidence in key actors was one of the factors that influences Malaysian public attitudes towards agro-biotechnology, suggesting that those who have a high level of confidence in key players tended to perceive the application as having higher risk acceptance, benefits, and encouragement [66]. Chen and Li showed that social trust in institutions is a predictor variable for perceived benefits and perceived risks [69], while Bronfman et al. also showed that trust in regulatory institutions by the public is positively associated with acceptability of hazards [78]. Therefore, trust in key players was included in this model and the following hypotheses were proposed.

H4: When the stakeholders have more trust in the key players involved in using or regulating modern biotechnology, then they perceive higher benefits associated with biodiesel.
H5: When the stakeholders have more trust in the key players involved in using or regulating modern biotechnology, then they will perceive a lower risk associated with biodiesel.
H6: When the stakeholders have more trust in the key players involved in using or regulating modern biotechnology, then they will have a more positive attitude towards biodiesel.

### Attitude towards technology

Impact of technologies is considered to be an important variable since it has been said to provide a picture of public views about biotechnology-related applications [72]. Earlier, Borcherding et al. developed a conceptual framework for the subjective evaluation of risky activities which hypothesised that the impact of technology is among the relevant co-determinants of risk perception variables [79]. A previous study of the Malaysian attitude towards biotechnology applications shows several general attitudes as predictors including a predisposition towards science and technology [71]. Respondents who have a negative predisposition towards science and technology were found to have more general concerns and viewed the product as being risky and having low benefits and encouragement. In this study, attitude towards technology consists of four items which reflect the negative impact brought by technology [66, 71]. A higher score of this construct indicates a higher negative predisposition towards science and technology from the respondent.

H7: When the stakeholders have a higher negative predisposition towards science and technology, they will then perceive a higher risk associated with biodiesel.

### Perceived risk and perceived benefit

Perceived benefits and perceived risks have been identified as important predictors in public attitude assessment [52, 72, 80–82]. Due to the complexity of the perception of risks and benefits, they are difficult to conceptualise separately, in fact few researchers believe that both the variables are not independent [72, 81]. Fischhoff et al. also reported a consistent association between perceived benefit and acceptable level of risk [83]. Perception of benefits revolves around producers, consumers, health, and societal issues, while risks are extended to include long-term effects on human health, the environment, and societal and moral issues [82]. Bredahl reported that public attitude towards technology is related to a weighing up of the perceived risks and perceived benefits [52]. If the perceived risks are more than the perceived benefits, consumers’ acceptance may be lower. Jensen and Andersen found a relationship between positive attitude towards biofuels and the benefits of biodiesel for climate and the environment [84].

However, the debate regarding the impact of biodiesel on food security around the world is also persistent. Many believe that a rapid growth in biodiesel production may take up precious agricultural land, affecting food security and subsequently influencing food prices [28, 29]. Previous studies have also claimed that food price increases have been mainly due to the expansion of biofuels [31, 32]. In contrast, several studies argued that the feedstock prices are irrelevant to the increase in biofuel production [85], suggesting that other factors including oil price developments and financial speculation may also contribute [34, 86]. Kallas and Gil previously found that consumers were not willing to pay for biodiesel if its production may negatively affect food prices [60].

Due to the importance of risk and benefit perception, the following hypotheses were proposed.

H8: When the stakeholders perceive higher risk associated with biodiesel, they will then perceive lower benefits associated with biodiesel.
H9: When the stakeholders perceive higher risk associated with biodiesel, then they will have a more negative attitude towards biodiesel.
H10: When the stakeholders perceive higher benefits associated with biodiesel, then they will have a more positive attitude to biodiesel.

## Research methodology

### Survey data collection

The research data were collected by means of a face-to-face survey of 509 adults aged 18-year old and above, residing in the Klang Valley region, Malaysia, from March 2012 to December 2012. The Klang Valley was chosen as the population of the study due to its role as the centre of Malaysia’s economic and social development. Furthermore, people residing in the area were from the diverse background required in this study. According to Krejcie and Morgan [87], for any population size beyond 5000, a sample size of 400 would be adequate, but there would be greater confidence with a sample of 500. Public acceptance of a new technology can be measured either by conducting a representative survey among lay people of a certain country, or by focusing on stakeholder representatives who contribute to the formation of public opinion and claim to represent certain public and private interests and concerns [88]. Most of the earlier research concentrated on representative public samples [72, 89–91], while other researchers, such as Aerni [88, 92], recommended the use of the stakeholder-based approach when it is difficult to run representative surveys in developing countries with low levels of public awareness of emerging technology.

In this study, the stakeholder-based survey approach recommended by Aerni [88, 92] was adopted but a wider range of interest groups were surveyed. Since the respective populations for the stakeholders involved were unknown, the respondents were chosen using the stratified purposive sampling technique as recommended by Monroe and Monroe [93]. This technique enables comparisons among respondents from different stakeholders group that might otherwise be underrepresented if random sampling were used. The respondents were stratified according to stakeholder groups which consisted of producers, scientists, policy makers, NGOs, media, religious scholars, university students, and consumers (general public). Each stakeholder group had a minimum target sample of 30 respondents. As recommended by Kelley, the questionnaires were handed over personally to the respondents by trained graduate enumerators [94]. Prior to completing the questionnaires, the respondents were first introduced to the basic concepts of modern biotechnology and the questionnaires were administered face-to-face. This approach was suggested by Kelley [94] to assess unsophisticated public attitudes to complex issues. It works perfectly well for both sophisticated respondents and unsophisticated respondents and allows the researchers to use sophisticated statistical multivariate procedures to discover whether the attitude responses are empirically sensible. By using a multiplicity of questions, measurement errors are reduced. There are several methods for surveys: face-to-face, telephone interviews and online. Each method has its own advantages and disadvantages. Face-to-face survey was chosen in this study for several reasons, such as higher response rate and better representation, as not all people have access to the internet and fixed telephone lines in Malaysia. Most people use mobile phones and numbers which are considered private and not available to outsiders. Telephone and online surveys have also been associated with avidity bias meaning that only people with a greater interest in the topic and the better educated with higher incomes will respond [95]. Although a survey carried out in the presence of the interviewer has been linked to social desirability bias, this issue is not consistent. Past studies have shown that only certain sensitive studies, such as those related to politics, sexual orientation or stigmatised behaviours have been recognised as subject to social desirability bias, otherwise there has been little evidence for mode effects [95, 96].

### Instrument

The multi-dimensional instrument measuring attitude towards biodiesel including the predicting factors was developed and adopted based on earlier studies. The instrument incorporated six variables, three of which were general dimensions, including engagement, trust in key players [72], and attitude towards technology [72, 97]. Three specific variables for the application included perceived risk [28, 97–99], perceived benefit [100], and overall attitude towards biodiesel [66, 72, 101]. All items were measured on 7-point Likert scales, except for engagement which was measured based on a total score of 10. Face and content validity of the instrument was determined by five experts in the area of attitude and perception studies, biotechnology and policy, environmental management, and measurement. As the questionnaires were developed in the Malay Language and included scientific terminologies, the language and terminologies were reviewed by two translation and science editorial experts to ensure that the terminologies and sentences could be understood by the public. The comments given by the experts were taken into account and the initial instrument was corrected. An English version of the instrument was also prepared and reviewed by the same translation and science editorial experts as some of the non-Malay respondents were not fluent in the Malay Language.

A pilot study of 200 respondents was undertaken to test the reliability and validity of the instrument. An exploratory principal component factor analysis followed by varimax rotation were carried out to identify items best expressive of attitudinal dimensions. The items which cross-loaded on more than two factors and were difficult to interpret, with factor loadings lower than 0.30 or inconsistency across the three modern biotechnology applications were deleted. The final instrument consisted of the following items. Engagement (*α* = 0.688) was measured by three items: past and intended behaviour, awareness, and knowledge. The first item was measured on a 7-point scale ranging from 1 (strongly disagree with the statement) to 7 (strongly agree with the statement), while the remaining two items were measured by a dichotomous nominal scale. Since they were all measured using a different scale, the items were later recoded so that they were valued out of 10 points. A higher score indicated greater engagement from the public.

Trust in key players (*α* = 0.838) was composed of the average mean response to the following three items: scientists have done a good job for society; producers have done a good job for society; and policy makers have done a good job for society. Each item was measured on a 7-point scale, ranging from 1 (strongly disagree with the statement) to 7 (strongly agree with the statement). A higher score indicated a greater confidence in the key players who are involved directly with the development of biotechnology in Malaysia.

Attitude towards technology (*α* = 0.875) was composed of four items: incessant science and technological progress will finally lead to humanity’s extermination; industry and technology have such an impact on urban life that you do not feel well and happy anymore; it is detrimental for humanity that the modern world is more and more dependent on technology; and modern technology has upset the balance of nature. Each item was measured on a 7-point scale, ranging from 1 (strongly disagree with the statement) to 7 (strongly agree with the statement). A higher score indicated a greater view on the impact of technology.

Perceived benefits (*α* = 0.654) was accessed by the following three items: to what extent is biodiesel useful to Malaysian society; biodiesel enables society to solve problems that currently cannot be solved by traditional methods; and the benefits of biodiesel exceed its risks. Each item was measured on a 7-point scale, ranging from 1 (not very useful/strongly disagree) to 7 (very useful/strongly agree). A higher score indicated greater perceived benefits of biodiesel.

Perceived risks (*α* = 0.696) was measured by four items: developing biodiesel will pose harm to the ecosystem and environment; biodiesel from crops may cause food shortages; biodiesel may lead to higher food prices; biodiesel may take up precious agricultural land, and considering all possible harms and adverse effects that might occur from developing and using biodiesel, how harmful are they? Each item was measured on a 7-point scale, ranging from 1 (strongly disagree/not worried at all/no harm at all) to 7 (strongly agree/very worried/very harmful). A higher score indicated greater perceived risks of biodiesel.

Attitude towards biodiesel (*α* = 0.896) was composed of five items: more intensive research should be encouraged to develop biodiesel; biodiesel should be scaled up/commercialised; the government should provide more financial support to researchers and industries in developing biodiesel; how far should biodiesel be encouraged; and the government is responsible to ensure biodiesel will benefit everyone. Each item was measured on a 7-point scale, ranging from 1 (not encouraged at all/strongly disagree/not willing at all) to 7 (strongly encouraged/strongly agree/very willing). A higher score indicated greater encouragement or overall attitude towards biodiesel.

### Statistical analysis

Initial reliability tests and confirmatory factor analysis of the pilot data were carried out using the International Business Machines (IBM) Statistical Package for the Social Sciences (SPSS) version 20 to assess the consistency and uni-dimensionality of the constructs. Correlation analyses were then undertaken at a bivariate level to determine the relationships among the variables which were hypothesised in the model (Fig. 1). A single-step SEM analysis, as recommended by Hair et al., was carried out on the final data to estimate the measurement and structural model using the IBM SPSS Analysis of a Moment Structures (AMOS) version 20 package with a maximum likelihood function [102, 103] as well as to determine the relationships between variables.

## Results and discussion

### Descriptive analysis

**Fig. 2:**
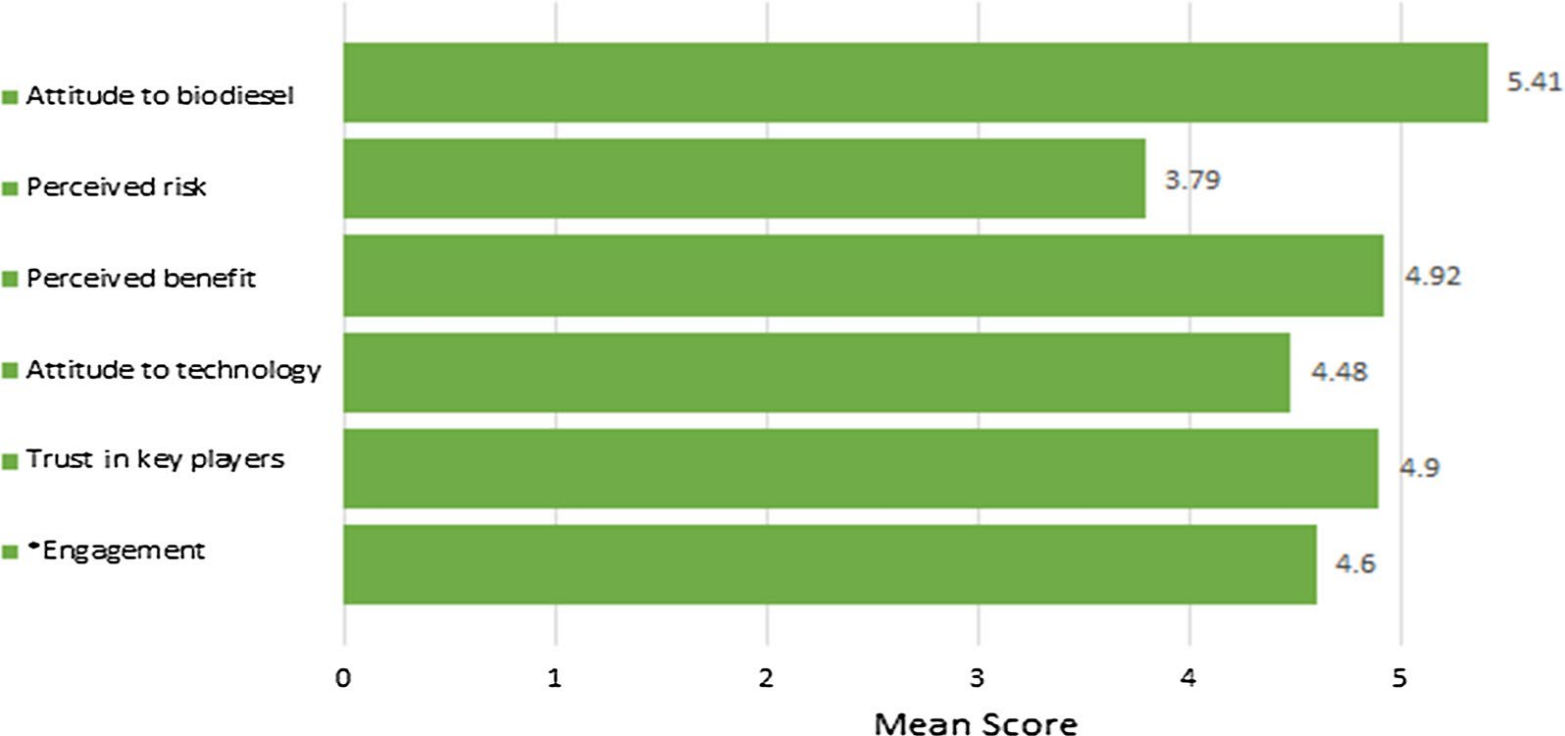
Attitude to biodiesel. 1–2.99: low, 3.00–5.00: moderate, 5.01–7.00: high. * 0–3.33: low, 3.34–6.66: moderate, 6.67–10: high

Before exploring the relationship between factors, the mean score for each variable was determined. In general, stakeholders in the Klang Valley region had a moderate mean score for all variables except for attitude towards biodiesel which exhibited a higher mean score (Table 3). Although both perceived benefits and perceived risks were categorised as moderate, the Malaysian stakeholders actually have seen more benefits of biodiesel (the mean score of perceived benefits is above the mid-point of 4.0), whereas they have seen lower risks and issues of food security associated with biodiesel (mean score below the mid-point of 4.0) (Table 3). A previous study confirmed that although a narrow majority was shown to support biofuels in general, the public/farmers tend to express greater enthusiasm about “second generation” of biofuels which do not involve edible crops [54], suggesting that the critical view from the public towards biofuel is food security and environmental issues. This result is also not surprising as the public were shown to be critical of biotechnology-related applications [66, 100, 104–106]. The Malaysian stakeholders, meanwhile, have also shown considerable trust in the key players as the mean score is significantly above the mid-point of 4.0 compared to other variables. Overall, the Malaysian stakeholders have expressed great support for biodiesel and believed it should be encouraged further, as indicated by the high mean score of attitude towards biodiesel (Fig. 2).

**Table 3:** Model comparison

Fit index	Model 1	Model 2	Model 3	Model 4
*χ* ^2^	504.6	508.6	380.6	353.6
*df*	196	199	179	180
*χ* ^2^/*df*	2.574	2.556	2.126	1.964
RMSEA (confidence interval)	0.056 (0.050–0.062)	0.055 (0.049–0.061)	0.047 (0.041–0.054)	0.044 (0.037–0.050)
GFI	0.914	0.913	0.935	0.939
AGFI	0.889	0.890	0.916	0.922
CFI	0.937	0.937	0.957	0.963
NFI	0.902	0.901	0.923	0.929
NNFI (TLI)	0.926	0.927	0.950	0.957

### Measurement model

Confirmatory factor analysis (CFA) was used to test the adequacy of the measurement model [107] and a total of six constructs were established. Correlation analysis was carried out at a bivariate level for each construct using the Pearson's correlation coefficient and the correlation matrix is presented in Table 2. The findings were used to develop the hypotheses presented in Fig. 1, thus constituting “model 1” of the attitude towards biodiesel in the structural equation modelling.

### Structural equation modelling

SEM is a powerful multivariate analysis technique where causal relationships among variables are sought [108–110]. SEM is capable of determining the relationships among a group of latent constructs which are represented by their measurements, as compared to other *general linear modelling* (GLM) [111]. In this study, a structural equation model was developed on the basis of previous research findings as well as correlations between the variables. A single-step SEM analysis, as recommended by Hair et al., was carried out to estimate the measurement and structural model using AMOS version 20 with a maximum likelihood function [103].

As recommended by Joreskog and Sorbom, the model generation strategy was used to specify the model, but the modifications of the nested models were only carried out when they were substantively meaningful [112]. A series of four nested models were tested to identify the best model for attitude towards biodiesel (Table 3). The first model was developed and specified according to the research framework described earlier (Fig. 1). It contained ten proposed hypotheses which were analysed to examine the relationship between the variables. It has been suggested that non-significant parameters should be removed from the original model and instead, additional paths suggested by the modification index added to improve the model fit as long as they were substantiated by the theory [113]. During model 1, 3 out of 10 hypotheses were eliminated due to being statistically insignificant at the probability level of 0.05. The changes were then saved as model 2. In model 2, the modification indices (MI) were observed to assure any potential pathway was included in the model; however, none of these pathways were suggested by the MI at this stage. In addition, standardised residual covariances for each item (observed variable) pair were observed, and those which had a value above 2.5 were considered for deletion [103]. Item 17 was found to have a number of high residual covariances with other items, therefore item 17 was deleted. The changes were then saved as model 3. The deletion of item 17 resulted in substantial changes among the model fit indicators as presented in Table 2. It also caused another two pathways to become statistically insignificant at the probability level of 0.05, leaving only 5 pathways in the model. Furthermore, correlated errors among the items in the same dimension were allowed [113]. Only one correlated error had been added into the model (between e7 of item 7 and e8 of item 8). The model was then named as model 4, and this model represents the final version of the model of Malaysian stakeholders’ attitude towards biodiesel. The summary of the results for hypothesis testing is listed in Table 4. Five hypotheses in Table 4 were supported (H1, H4, H7, H8, H10), indicating that the related hypothesised paths were found to be significant while the remaining five hypotheses were rejected (H2, H3, H5, H6, H9), reflecting insignificant relationships between the associated factors.

**Table 4:** Hypothesis results for the structural model

Research hypothesis		Conclusion
H1	Engagement → perceived benefit	Supported
H4	Trust in key players → perceived benefit	Supported
H7	Attitude towards technology → perceived risk	Supported
H8	Perceived risk → perceived benefit	Supported
H10	Perceived benefit → attitude towards biodiesel	Supported
H2	Engagement → perceived risk	Not supported
H3	Engagement → attitude towards biodiesel	Not supported
H5	Trust in key players → perceived risk	Not supported
H6	Trust in key players → attitude to biodiesel	Not supported
H9	Perceived risk → attitude towards biodiesel	Not supported

A well-fitting model should have GFI, AGFI, and CFI greater than 0.90 while the RMSEA value was less than 0.05 supported with a narrow confidence interval [114, 115]. Other than these three fit indices, Costa-Font and Gil also used several commonly used fit indices to assess the overall model fit. These included Chi square (*χ* ^2^), CMIN/DF (*χ* ^2^/*df*), NFI, and NNFI [116]. Carmines and McIver suggested that a good model should have a value of *χ* ^2^/*df* less than 3 [117]. The measurement model for the final version of the model of attitude towards biodiesel was found to have a good fit with CMIN/DF = 1.964, CFI = 0.963, GFI = 0.939, and RMSEA = 0.044. The fit indexes for each model during model development under the structural equation modelling technique are presented in Table 3.

### Construct reliability and validity

The three types of reliabilities measured in this paper were the internal consistency (Cronbach alpha), item reliability, and construct reliability. The Cronbach’s alpha coefficients for all constructs were above 0.65 and were considered to be good (Table 5). The item reliability, which is presented by corrected item-total correlations for items in each dimension, was also considered to be good. All correlation coefficients were greater than 0.5 except for “past and intended behaviour” (0.469) under engagement; “developing biodiesel will pose harm to the ecosystem and environment” (0.485); “to what extent is biodiesel useful to the Malaysian society?” (0.491); and “biodiesel enables the society to solve problems that currently cannot be solved by traditional methods” (0.481) (Table 5). The construct reliability was represented by the composite reliabilities and the average variance extracted (AVE). From Table 5, the composite reliabilities for all constructs are above 0.7 while the variance extracted (AVE) are above 0.45 indicating good construct reliability [103].

**Table 5:** Measurement scales, reliability, and validity of public perception towards biodiesel

Factor and item	Corrected item-total correlated	*α*	Standardised factor loading	Composite reliability	Average variance extracted (AVE)
Engagement		0.688		0.715	0.459
1. Past and intended behaviour	0.469		0.571		
2. Awareness	0.567		0.712		
3. Knowledge	0.523		0.737		
Trust in key players		0.838		0.841	0.638
4. Scientists have done a good job for society	0.657		0.749		
5. Producers have done a good job for society	0.737		0.847		
6. Policy makers have done a good job for society	0.709		0.798		
Attitude towards technology		0.875		0.864	0.614
7. Lead to humanity’s extermination	0.704		0.692		
8. Impact on urban life	0.786		0.788		
9. Detrimental to humanity	0.753		0.862		
10. Upset the balance of nature	0.689		0.784		
Perceived benefit		0.713		0.816	0.465
11. Useful to the society	0.491		0.708		
12. Solve problems that currently cannot be solved by traditional method	0.481		0.570		
13. Benefits exceed risks	0.631		0.754		
Perceived risk		0.677		0.733	0.509
14. Pose harm to the ecosystem and environment	0.485		0.438		
15. May cause food shortage	0.651		0.906		
16. May lead to higher food price	0.569		0.769		
Attitude to biodiesel		0.896		0.897	0.636
18. More intensive research should be encouraged	0.693		0.743		
19. Should be scaled up/commercialised	0.755		0.807		
20. Should be given monetary support by government	0.773		0.823		
21. Government responsibility to assure the product is beneficial	0.768		0.810		
22. Overall encouragement	0.737		0.803		

### Relationships among the constructs

**Fig. 3:**
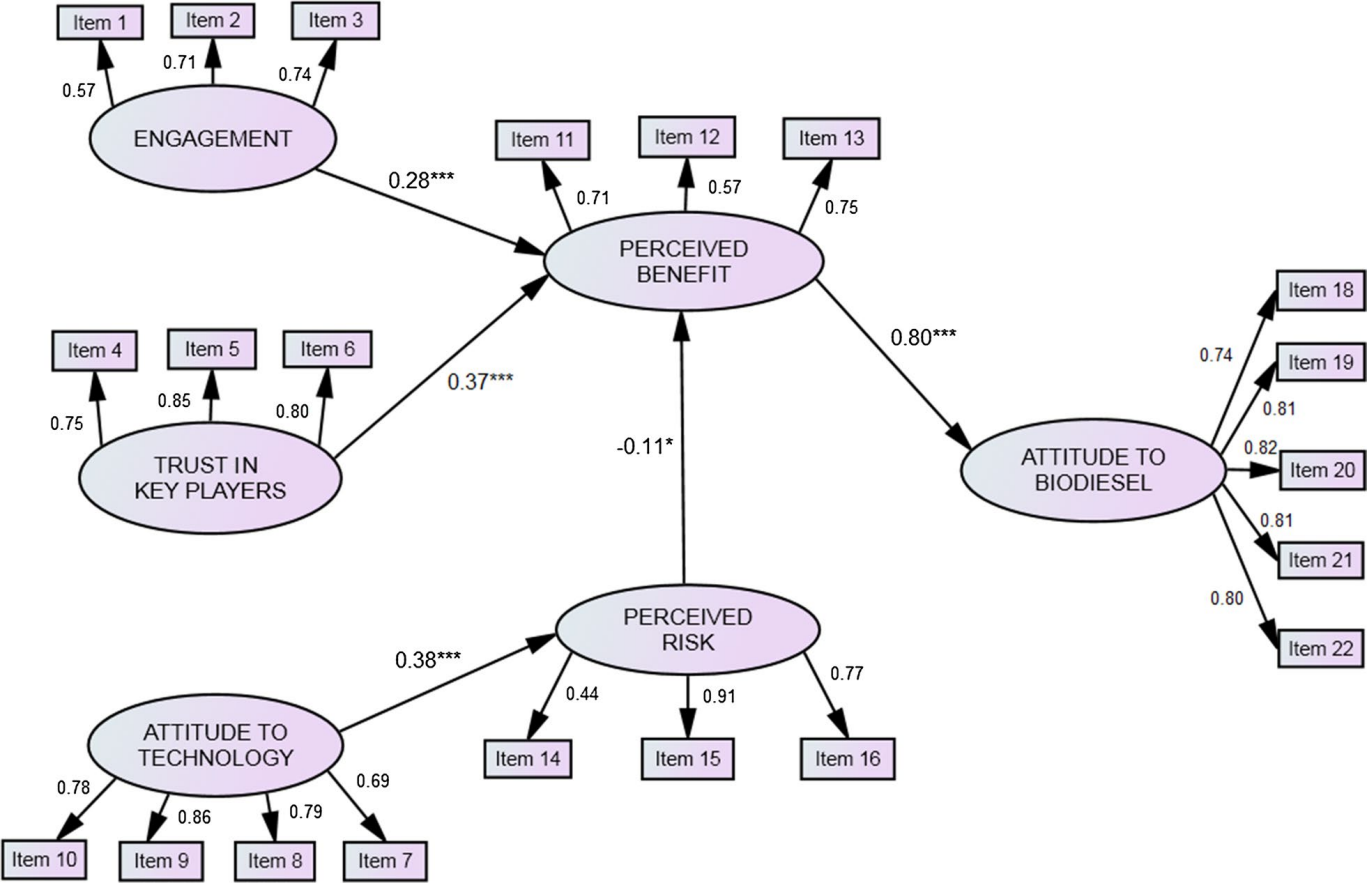
Structural equation model of factors influencing stakeholders’ attitude towards biodiesel showing interrelationships among variables. Standardised estimates are presented. *p < 0.05, **p < 0.01, ***p < 0.001. Items description can be found in Table 5

Figure 3 shows the final structural model of Malaysian stakeholders’ attitudes towards biodiesel. Perceived benefit emerged as the most important direct predictor of the attitude towards biodiesel (*β* = 0.80, *p* < 0.001) (Fig. 3), suggesting that the Malaysian stakeholders have strongly considered the beneficial aspects of biodiesel when expressing their support of the application. This finding is supported by the earlier study where perceived benefit was also found to be the main predictor of attitude towards genetically modified soybean [66], GM mosquito [118], and GM rice [119]. Gaskell et al. previously found perceived benefits as the pre-condition of support for several biotechnology applications [72], while Jensen and Andersen illustrated that a positive attitude towards biofuel exists when respondents perceive them as beneficial for the climate and environment [84]. In a Greek study, Savvanidou et al. also demonstrated a critical view by the public regarding their acceptance of biofuels in relation to perceived benefits. Despite moderate responses that “the use of biofuels can be an effective solution against climatic changes” and “biofuels can be an effective solution for the energy problem”, more than 80% of the respondents were still willing to use biofuels [120].

Other than perceived benefit, stakeholders’ attitudes towards biodiesel also involved the interplay between other factors, such as engagement to biotechnology, trust in key players, attitude to technology, and perceived risk. The results of the study agreed with previous findings which suggest that an inverse relationship exists between perceived risk and perceived benefit of the biotechnology products [63, 66, 118, 121, 122]. Perceived risk has been found to have a negative association with perceived benefit (*β* = −0.11, *p* < 0.05), implying that the public who view biodiesel as a potential threat to food security, as well as giving rise to its indirect environmental impact, will tend to perceive its promises as low, and vice versa. In this study, any justification regarding food security issues surrounding biodiesel by the public may be strongly incorporated with other forms of risk of the application (to form perceived risk), for instance, concern about the issues of the environment and biodiversity due to its development. Previous literature has discussed the issue of food security as a separate issue rather than combining it with other risks or treating them as a collective issue [28, 29, 31, 32]. It is believed that rapid growth in biodiesel production may take up precious agricultural land, as well as affect the LUC of the area. Furthermore, this action may have a direct impact on food security and subsequently influence food price increases [28, 29]. Previous studies claimed that food price increases have principally been due to the expansion of biofuels [31, 32]. However, several studies also argued that the feedstock prices are irrelevant to the increase in biofuel production [85], suggesting that other factors, including oil price developments and financial speculation, may be relevant [34, 86].

The model also suggests the role of perceived benefit as a mediator between engagement and trust in key players in the attitude towards biodiesel. Those who are more engaged and “informed” (aware and knowledgeable) about biotechnology will perceive higher benefits associated with biodiesel (*β* = 0.28, *p* < 0.001) and, consequently, they will encourage the product further. This finding also highlights the importance of knowledge and awareness along with past and intended behaviour, in order to develop positive attitudes towards biodiesel, including perceived benefit, as the public only form attitudes about technologies after they have acquired relevant information [94]. However, Cacciatore et al. found that more knowledgeable respondents see fewer benefits of biofuels relative to risks. This finding could be moderated by the effect of partisanship (political views) [56].

This finding also suggests that the level of trust in key players has influenced the perceived benefit of biodiesel positively (*β* = 0.37, *p* < 0.001) and could be key to driving the public to encourage the product further. This indicates that if the respondents had more confidence in the key players involved in biotechnology, such as the scientists, producers, and policy makers, they would perceive biodiesel as beneficial. This finding is in parallel with the research of Amin et al. who also found a significant association exists between the confidence in key players and the perceived benefit [66, 118]. Trust in key players, such as the government and scientists, is considered to be an important determinant of positive attitude towards GM technology in previous literature [72, 123, 124]. Enforcing a specific regulatory control on biodiesel practice will not only enhance the public confidence in policy makers and the industry, but would also create a good impression of the quality and capacity of the product, which in turn influences the public to support the product. For instance, the imposition of sustainability criteria in the 2009 Renewable Energy Directive by the European Union was a necessary condition for public and parliamentary support for renewable fuel mandates [125]. In addition, through the Air Pollution Control (Motor Vehicle Fuel) Regulation 2009, the Hong Kong government have set a mandatory requirement that motor vehicle biodiesel must follow certain international standards, such as EN 14214 in order to boost consumer confidence in the fuel and prevent excessive exhaust emissions due to poor biodiesel quality [126]. The regulations have also highlighted that the fuel must be labelled at the point of sale if the biodiesel content in motor vehicle fuel exceeds 5% [126].

The attitude towards technology has been found to have a positive association with perceived risk (*β* = 0.38, *p* < 0.001), implying that those who hold a negative predisposition toward technology were more likely to perceive a higher risk associated with biodiesel production. The public view on modern technology that it is disrupting the balance of nature could have influenced their agreement about the negative impact of biodiesel on the basis that it poses harm to the ecosystem and environment. These relationships are also supported by previous findings, where respondents who have a negative predisposition towards science and technology were found to have more general concerns and viewed the application as unfamiliar, risky, and having higher moral concerns [71, 118].

### Limitations of the study and suggestions

There are a number of limitations to this study. The focus of this study was stakeholders’ attitudes to biodiesel. The predicting factors in our model included those that had been proposed by previous studies related to attitude to biotechnology. However, the number of predictors was not exhaustive. Nevertheless, the findings of this study provide an important insight from the perspective of various stakeholders, despite the limited coverage, as well as highlights the Asian view of biodiesel. In future, there is a need for a study which focuses on stakeholders’ willingness to buy/use biodiesel. This should include economic factors, such as fuel price rises and vehicle compatibility as well as identify their contribution to stakeholders’ willingness to use/adopt biodiesel. In this study, stakeholders were asked about their attitude to biodiesel derived from crops such as palm oil and its use as biofuel for vehicles. There is also a need for future studies to assess stakeholders’ perceptions about the second, third, and fourth generation of biofuels. Malaysia is a multi-ethnic country, so it is important to analyse the effect of ethnicity and religion, as well as other demographic variables, such as stakeholder groups, age, education, and gender, on attitude to biodiesel. In future, we will undertake further analysis of the data and write another article on this important area of research.

## Conclusions

Structural equation modelling has confirmed that the Malaysian stakeholders’ attitudes towards the use of biodiesel derived from crops, such as palm oil, as biofuel for vehicles is a complex matter which should be seen as a multi-faceted process. The most important direct predictor for attitudes towards biodiesel is the specific application-linked perceptions of their benefit, followed by trust in key players. This indicates that the Malaysian stakeholders tend to focus not only on the beneficial aspects of biodiesel, but also on their trust in the industry, scientists, and policy makers. These may become the pre-conditions for their support of the use of biodiesel for vehicles. Stakeholders’ attitudes towards biodiesel also involve the interplay between other factors such as perceived risk, engagement, and attitude towards technology. The findings of this research serve as principal information regarding public opinion and attitudes towards the development and commercialisation of biodiesel in this region. It is very useful to observe and understand the findings presented in this paper in order to understand the social acceptance of biodiesel in a highly developing country such as Malaysia. Understanding the matter is crucial when embarking on related research and commercialisation, in order to avoid the loss of significant financial and labour investments if biodiesel products are unacceptable to consumers in the future.
